# Effect of Adenotonsillectomy on Serum Levels of Total Antioxidant-A Prospective Cohort Study

**Published:** 2019-05

**Authors:** Mohammad Saeid Ahmadi, Elham Tayebi, Hamid Mazarei, Javaneh Jahanshahi

**Affiliations:** 1 *Department of Otolaryngology Head and Neck Surgery, Hamadan University of Medical Science, Hamadan, Iran. *

**Keywords:** Adenotonsillectomy, Antioxidants, Free radicals

## Abstract

**Introduction:**

Antioxidants are essential elements in reducing the harmful effects of free radicals. The aim of this study was to compare the serum levels of total antioxidant before and after adenotonsillectomy procedure.

**Materials and Methods:**

This study was conducted on 41 children with the age range 3-12 who underwent adenotonsillectomy. The total level of antioxidants was measured before and two months after adenotonsillectomy.

**Results:**

The mean±SD level of serum antioxidants raised significantly from 11.17 (15.36) before surgery to 22.19 (10.87) IU/mL after surgery (P<0.001). The mean value was increased from 15.50 (17.97) to 24.89 (12.66) and 6.62 (10.67) to 19.36 (7.98) IU/ml among males and females, respectively.

**Conclusion:**

Adenotonsillectomy can efficiently increase the serum levels of total antioxidant among children with adenotonsillar hypertrophy. Moreover, it can improve the immune system against free radicals.

## Introduction

Recurrent tonsillitis and adenoids hypertrophy problems are the most common complaints in pediatric practice. Tonsillitis and adenoid diseases involve a wider range of symptoms, such as snoring, open -mouth breathing, fatigue, and obstructive sleep apnea (1). In addition, it may lead to recurrent pharyngitis, fever, dysphagia, malaise, and nocturnal enuresis (2). The practicing otolaryngologists and other practitioners, such as pediatrics, dentists, and orthodontists, benefit from the definition, classification, pathophysiology, evaluation, and treatment methods of adenotonsillar hypertrophy and obstructive sleep apnea. 

There are several kinds of activated molecules of oxygen species, such as hydroxyl radicals, superoxide anions, and hydrogen peroxides, which are the products of active granulocytes. These species act important roles in a lot of biochemical processes, such as intracellular messaging, apoptosis, and immunity. On the other hand, excessive production of these reactive oxygen species, which may be present during the inflammation process results in oxidative stress (3).

Oxidative metabolism is created within the cell in different cellular interactions. Chemical materials, drugs, X radiation, cellular aging, and phagocytes, may cause the production of oxidant molecules. They should be metabolized by antioxidants; otherwise, they may react with cellular proteins and DNAs leading to cellular injury and death (4). 

Antioxidants are well-known elements that interact with the oxidants and neutralize their harmful effects at the molecular level. Moreover, they play an important role in the treatment of oxidant-related disorders (5). Given the important role of antioxidants in the human body, this study aimed at measuring serum levels of total antioxidant before and after adenotonsillectomy among children who underwent this procedure.

## Materials and Methods

This study was conducted in 2016 (April to September) on 41 children who referred to Besat Hospital, Hamadan, Iran, and were candidates for adenotonsillectomy based on the history and physical examination. The study protocol was approved by the Ethics Committee of Hamadan University of Medical Sciences, Hamadan, Iran. The patients who were diagnosed with chronic tonsillitis and adenoid hypertrophy based on the history and physical examination were enrolled in the study. In addition, the children with systemic disease or previous history of adenoidectomy were excluded from the study. 

After obtaining informed consent from parents, blood samples (5 ml) were taken from patients a day before surgery. The serum was separated from the blood cells (1500 rpm, 10-15 min) in order to analyze the serum levels of total antioxidant using enzyme-linked immunosorbent assay (ELISA). 

Subsequently, the samples were stored at -20 ^°^C. Adenotonsillectomy was performed using cold dissection and routine curettage methods under general anesthesia. Two months after the operation, blood samples were retaken from the children in a similar way for the same analysis.

It should be noted that the participants were not involved with infectious diseases nor exposed to X radiation or used drugs one month before first sampling and during two months after the surgery. 

The biochemical measurements were done in the same laboratory using Human Total Antioxidant Capacity, ELISA kit, Glory science Co., Ltd. The data were analyzed in Stata software (Version 11) (Stata Corp, College Station, TX, USA) through t-test to compare the test results before and after the surgery. In addition, p-value less than 0.05 was considered statistically significant. 

## Results

This study was conducted on 41 children with the age range and mean±SD age of 3-12 and 6.41(2.44) years, respectively. Out of total patients, 21 cases were males. Table 1 presents the mean level of serum antioxidants before and after adenotonsillectomy. 

According to the results, there was a significant increase in the mean level of postoperative serum antioxidants, compared to the one before surgery (22.19 vs. 11.7 IU/ml, P<0.001). The level of preoperative serum antioxidant increased from 15.50(17.97) to 24.89(12.66) and 6.62(10.67) to 19.36 (7.98) among males and females, respectively (Table.1).

**Table 1:** Comparison of serum levels of total antioxidant (IU/mL) before and after adenotonsillectomy

		**Before tonsillectomy**		**After tonsillectomy**		
**Variable**	**Number**	**Mean**	**SD**	**Mean**	**SD**	**P-value**
Overall	41	11.17	15.36	22.19	10.87	0.001
Males	21	15.50	17.97	24.89	12.66	0.001
Females	20	06.62	10.67	19.36	07.98	0.001

## Discussion

Acute tonsillitis and adenotonsillar hypertrophy are the most common complaints in pediatrics (4). Palatine tonsils are the first line of defense against upper respiratory tract infections. Recurrent exposure to external pathogens contributes to chronic inflammation that could be characterized by tonsillar hypertrophy and sclerosis of tonsillar structures among children and adults, respectively (6). Many studies have shown the association between the recurrent inflammations and the production of free oxidative radicals. Moreover, several studies revealed an increase in serum levels of antioxidant after adenotonsillectomy. This result was in line with the findings in this study. 

The oxidative metabolism of peripheral blood granulocytes is detected in 30% of patients with tonsillar hypertrophy and 75-90% of cases who suffered from recurrent tonsillitis (7). Because of the high reactiveness of these molecules, they can cause some degrees of harmful effects to tissues, especially due to reaction with cellular lipids, proteins, nucleotides, and carbohydrates, in cell membranes.

In the normal balanced circulation, the harmful effects of these molecules are deactivated with antioxidants in the human body (8). Low levels of antioxidants in serum could have negative effects on children immune system which expose them to recurrent upper respiratory infections (9). 

Kaygusuz et al. conducted a study on 124 patients who underwent tonsillectomy. They measured malondialdehyde and superoxide dismutase levels before and two weeks after surgery and showed the important effect of oxidative stresses on the pathogenicity of recurrent tonsillitis (3). 

Yılmaza et al. determined the remarkable role of oxidants and antioxidants in the pathogenicity of recurrent tonsillitis and adenoid hypertrophy (10). Moreover, Dugruer et al. showed significantly higher levels of malondialdehyde as well as increased activities of superoxide dismutase and glutathione peroxidase tonsillectomy procedure. However, no significant differences were observed between the preoperative and postoperative periods in terms of catalase activity. They revealed that the defense mechanisms of oxidants and antioxidants were promoted after tonsillectomy (11). 

In a study conducted by Kiroglu et al., the possible act of oxidants and antioxidants in the pathogenicity of chronic and recurrent adenotonsillitis was determined among children. They confirmed that activated oxidative and antioxidants had significant effects on the pathogenicity of adenotonsillar hypertrophy and chronic adenotonsillitis (12).

Another study found that oxidative stress might still present a month after tonsillectomy among patients who suffered from tonsillar hypertrophy and recurrent tonsillitis; however, it decreased after adenotonsillectomy (6). 

Recent studies demonstrated a significant increase in antioxidants level followed by a decrease in oxidative stress after adenotonsillectomy. The obtained results are consistent with the findings in the literature and the present study (13,14). Furthermore, adenotonsillectomy could remove microbial sources among children with recurrent adenotonsillar infections leading to decreased general oxidative stress. 

Despite the increase in antioxidant levels after adenotonsillectomy, no normal level was observed in this study because of the short period follow up that seems to be the most important limitation of the current study. A two-month follow-up period was probably not long enough for the normalization of oxidative products. 

Due to the signiﬁcant postoperative rising of the antioxidants levels, it is revealed that adenotonsillectomy is helpful in such a context. The strengths of similar studies and this study are platforms for future studies. As it is mentioned earlier, because of the importance of long-term follow-up of patients, it is suggested that further studies consider longer follow-ups with larger sample sizes.

## Conclusion

Adenotonsillectomy can significantly increase serum levels of total antioxidant after the surgery among children with adenotonsillar hypertrophy. 

## References

[1] Shirley WP, Woolley AL, Wiatrak BJ, FLint PW, Haughey BH, Lund VJ, Niparko JK, Richardson MA, Robins KT (2015). Pharyngitis and adenotonsillar disease. Cummings otolaryngology head & neck surgery.

[2] Ahmadi MS, Amirhassani S, Poorolajal J (2013). The effect of adenotonsillectomy on pediatric nocturnal enuresis: a prospective cohort study. Iranian Journal of Otorhinolaryngology.

[3] Kaygusuz I, Ilhan N, Karlidag T, Keles E, Yalcin S, Cetiner H (2003). Free radicals and scavenging enzymes in chronic tonsillitis. Otolaryngology head and neck surgery: official journal of American Academy of Otolaryngology-Head and Neck Surgery.

[4] Yilmaz T, Kocan EG, Besler HT (2004). The role of oxidants and antioxidants in chronic tonsillitis and adenoid hypertrophy in children. Int J Pediatr Otorhinolaryngol.

[5] Kalayci O, Besler T, Kilinc K, Sekerel BE, Saraclar Y (2000). Serum levels of antioxidant vitamins (alpha tocopherol,beta carotene,and ascorbic acid) in children with bronchial asthma. The Turkish journal of pediatrics.

[6] Cvetkovic T, Vlahovic P, Todorovic M, Stankovic M (2009). Investigation of oxidative stress in patients with chronic tonsillitis. Auris, nasus, larynx.

[7] Kowalska M, Kowalska H, Zawadzka-Glos L, Debska M, Szerszen E, Chmielik M (2003). Dysfunction of peripheral blood granulocyte oxidative metabolism in children with recurrent upper respiratory tract infections. Int J Pediatr Otorhinolaryngol.

[8] Shukla GK, Garg A, Bhatia N, Pandey S, Kaur G, Shukla RN (2000). Significance of free radicals in chronic tonsillitis. Bollettino chimico farmaceutico.

[9] Helmut S, physiology E (1997). Oxidative stress: Oxidants and antioxidants. Exp Physiol.

[10] Yılmaza T, Koçana EG, Besler HT (2004). The role of oxidants and antioxidants in chronic tonsillitis and adenoid hypertrophy in children. Int J Pediatr Otorhinolaryngol.

[11] Dogruer ZN, Unal M, Eskandari G, Pata YS, Akbas Y, Cevik T (2004). Malondialdehyde and antioxidant enzymes in children with obstructive adenotonsillar hypertrophy. Clinical biochemistry.

[12] Kiroglu AF, Noyan T, Oger M, Kara T (2006). Oxidants and antioxidants in tonsillar and adenoidal tissue in chronic adenotonsillitis and adenotonsillar hypertrophy in children. Int J Pediatr Otorhinolaryngol.

[13] Shishegar M, Ashraf AM (2015). Assessment of oxidant and antioxidant serum levels in children with chronic tonsillitis before and after tonsillectomy. Indian journal of basic and applied medical research.

[14] Hoon Cho J, D Sub J, WonKim Y, Hong S, Kook Kim J (2015). Reduction in oxidative stress biomarkers after adeno tonsillectomy. International journal of pediatric otorhinolaryngology september.

